# Draft Genome Sequence of Haloferax volcanii SS0101, Isolated from Salt Farms in Samut Sakhon, Thailand

**DOI:** 10.1128/MRA.00926-19

**Published:** 2019-11-14

**Authors:** Suwanan Wanthongcharoen, Wariya Yamprayoonswat, Pattarawan Ruangsuj, Nuttida Thongpramul, Watthanachai Jumpathong, Satapanawat Sittihan, Pornpimon Kanjanavas, Montri Yasawong

**Affiliations:** a Program on Environmental Toxicology, Chulabhorn Graduate Institute, Chulabhorn Royal Academy, Bangkok, Thailand; b Center of Excellence on Environmental Health and Toxicology (EHT), Office of Higher Education, Bangkok, Thailand; c Program on Chemical Biology, Chulabhorn Graduate Institute, Chulabhorn Royal Academy, Bangkok, Thailand; d Division of Biological Science, Faculty of Science and Technology, Huachiew Chalermprakiet University, Samut Prakan, Thailand; Portland State University

## Abstract

Haloferax volcanii SS0101 is a halophilic archaeon isolated from salt farms in Thailand. The genome sequence of H. volcanii SS0101 contains a gene encoding capreomycidine synthase, a key enzyme for capreomycidine biosynthesis. This 3.8-Mb draft genome sequence of H. volcanii SS0101 will provide the tools for investigating genes involved in capeomycidine production in haloarchaea.

## ANNOUNCEMENT

Haloferax volcanii is an extremely halophilic archaeon (1–3) and aerobic chemoorganotroph (1). Cells of H. volcanii are Gram negative, pleomorphic, and mostly flattened or disk shaped (1). The optimal growth temperature of H. volcanii is 35°C (1). The salt concentration for optimal growth is approximately 2.5 M (1). The major polar lipid components of H. volcanii are phosphatidylglycerophosphate (PGP) and sulfated diglycosyl diether (S-DG) (1). H. volcanii is an important cellular model system for the genetic, molecular biological, and biochemical study of archaea (4).

Strain SS0101 was isolated from soil samples collected from salt farms (13°30′47ʺN, 100°22′29ʺE) in Samut Sakhon, Thailand. One gram of soil was dissolved in phosphate buffer containing 3.4 M NaCl, and the extinction dilution method was performed to isolate strain SS0101. Strain SS0101 was grown in JCM169 broth containing 3.4 M NaCl at 37°C with shaking at 250 rpm. Genomic DNA (gDNA) of strain SS0101 was extracted with the phenol chloroform method described by Sambrook and Russel (5). The quality and quantity of gDNA were determined using a NanoDrop spectrophotometer (Thermo Fisher Scientific, USA). The sequencing library was prepared using the Ion Plus fragment library kit and the Ion PI Hi-Q OT2 200 template kit. The sequencing was performed on the Ion Proton sequencer using the Ion PI Hi-Q sequencing 200 kit and the Ion PI chip (Thermo Fisher Scientific, USA). The average read length was 101 bp. There were 5,993,233 raw reads (155× depth of coverage) generated from the sequencing run. The quality of the raw reads was determined using AfterQC 0.9.6 with default parameters (6). *De novo* genome assembly of the raw reads was performed with SPAdes 3.13.1 in the careful mode (7). The genome was decontaminated using Automated Contamination Detection and Confidence (ACDC) 1.02 with default parameters (8). The genome assembly metrics of the decontaminated contigs of H. volcanii SS0101 were determined using QUAST 5.0.2 with default parameters (9). The draft genome sequence of H. volcanii SS0101 consists of 3,784,342 bp in 593 contigs with an *N*_50_ value of 18,910 bp and a 66.08% G+C content. Gene prediction was performed using Prokka 1.13.7 with default parameters (10). The annotated genome sequence of H. volcanii SS0101 contains 3,853 protein-coding sequences along with 51 tRNAs, 1 rRNA operon, and 7 CRISPR regions.

The draft genome sequence of strain SS0101 was identified using the Genome-to-Genome Distance Calculator (GGDC) 2.1 with default parameters (11, 12). The archaeal strain SS0101 was affiliated with Haloferax volcanii DS2 (GenBank accession number CP001956) with an 80.6% supported value of the digital DNA-DNA hybridization (dDDH). The difference in G+C content between strain SS0101 and H. volcanii DS2 (CP001956 ) was 0.56%.

There is one annotated locus of the capreomycidine synthase gene in the H. volcanii SS0101 genome. Capreomycidine synthase is a key enzyme for capreomycidine synthesis. Capreomycidine is a nonproteinogenic amino acid, an important residue found in the tuberactinomycin family of antitubercular peptide antibiotics (13).

### Data availability.

The whole-genome shotgun sequence of Haloferax volcanii SS0101 has been deposited at DDBJ/ENA/GenBank under the accession number VMTR00000000 and SRA number SRR9831208.

## References

[1] Torreblanca M, Rodriguez-Valera F, Juez G, Ventosa A, Kamekura M, Kates M 1986 Classification of non-alkaliphilic halobacteria based on numerical taxonomy and polar lipid composition, and description of *Haloarcula* gen. nov. and *Haloferax* gen. nov. Syst Appl Microbiol 8:89–99. doi:10.1016/S0723-2020(86)80155-2.

[2] Euzéby JP, Kudo T 2001 Corrigenda to the validation lists. Int J Syst Evol Microbiol 51:1933–1938. doi:10.1099/00207713-51-5-1933. 11594628

[3] International Journal of Systematic and Evolutionary Microbiology. 1986 Validation of the publication of new names and new combinations previously effectively published outside the IJSB. List no. 22. Int J Syst Bacteriol 36:573–576. doi:10.1099/00207713-36-4-573.

[4] Pohlschroder M, Schulze S 2019 Haloferax volcanii. Trends Microbiol 27:86–87. doi:10.1016/j.tim.2018.10.004. 30459094

[5] Sambrook J, Russell RW 2001 Preparation and analysis of eukaryotic genomic DNA, molecular cloning: a laboratory manual, 3rd ed, vol 1 Cold Spring Harbor Laboratory Press, Cold Spring Harbor, NY.

[6] Chen S, Huang T, Zhou Y, Han Y, Xu M, Gu J 2017 AfterQC: automatic filtering, trimming, error removing and quality control for fastq data. BMC Bioinformatics 18:80. doi:10.1186/s12859-017-1469-3. 28361673 PMC5374548

[7] Nurk S, Bankevich A, Antipov D, Gurevich AA, Korobeynikov A, Lapidus A, Prjibelski AD, Pyshkin A, Sirotkin A, Sirotkin Y, Stepanauskas R, Clingenpeel SR, Woyke T, McLean JS, Lasken R, Tesler G, Alekseyev MA, Pevzner PA 2013 Assembling single-cell genomes and mini-metagenomes from chimeric MDA products. J Comput Biol 20:714–737. doi:10.1089/cmb.2013.0084. 24093227 PMC3791033

[8] Lux M, Krüger J, Rinke C, Maus I, Schlüter A, Woyke T, Sczyrba A, Hammer B 2016 ACDC: automated contamination detection and confidence estimation for single-cell genome data. BMC Bioinformatics 17:543. doi:10.1186/s12859-016-1397-7. 27998267 PMC5168860

[9] Mikheenko A, Prjibelski A, Saveliev V, Antipov D, Gurevich A 2018 Versatile genome assembly evaluation with QUAST-LG. Bioinformatics 34:i142–i50. doi:10.1093/bioinformatics/bty266. 29949969 PMC6022658

[10] Seemann T 2014 Prokka: rapid prokaryotic genome annotation. Bioinformatics 30:2068–2069. doi:10.1093/bioinformatics/btu153. 24642063

[11] Meier-Kolthoff JP, Auch AF, Klenk H-P, Göker M 2013 Genome sequence-based species delimitation with confidence intervals and improved distance functions. BMC Bioinformatics 14:60. doi:10.1186/1471-2105-14-60. 23432962 PMC3665452

[12] Meier-Kolthoff JP, Göker M, Klenk H-P 2014 Taxonomic use of DNA G+C content and DNA-DNA hybridization in the genomic age. Int J Syst Evol Microbiol 64:352–356. doi:10.1099/ijs.0.056994-0. 24505073

[13] Fei X, Yin X, Zhang L, Zabriskie TM 2007 Roles of VioG and VioQ in the incorporation and modification of the capreomycidine residue in the peptide antibiotic viomycin. J Nat Prod 70:618–622. doi:10.1021/np060605u. 17302456 PMC2825577

